# Ferric carboxymaltose reduces transfusions and hospital stay in patients with colon cancer and anemia

**DOI:** 10.1007/s00384-015-2461-x

**Published:** 2015-12-22

**Authors:** José Luis Calleja, Salvadora Delgado, Adolfo del Val, Antonio Hervás, José Luis Larraona, Álvaro Terán, Mercedes Cucala, Fermín Mearin, on behalf of the Colon Cancer Study Group

**Affiliations:** Digestive Diseases Department, Hospital Puerta de Hierro, Calle Manuel de Falla 1, Majadahonda, 28222 Madrid Spain; Gastrointestinal Surgery Department, Hospital Clínic, Barcelona, Spain; Digestive Diseases Department, Hospital La Fe, Valencia, Spain; Digestive Diseases Department, Hospital Reina Sofía, Córdoba, Spain; Digestive Diseases Department, Hospital Nuestra Señora de Valme, Sevilla, Spain; Digestive Diseases Department, Hospital Marqués de Valdecilla, Santander, Spain; Medical Department, Vifor Pharma España, Barcelona, Spain; Digestive Diseases Department, Centro Médico Teknon, Barcelona, Spain

**Keywords:** Iron deficiency anemia, Colon cancer surgery, Iron intravenous administration, Ferric carboxymaltose

## Abstract

**Purpose:**

The purpose of the study was to evaluate the efficacy of preoperative intravenous (IV) ferric carboxymaltose (FCM) administration vs. no-IV iron in colon cancer (CC) anemic patients undergoing elective surgery with curative intention.

**Methods:**

This was a multicenter, observational study including two cohorts of consecutive CC anemic patients: the no-IV iron treatment group was obtained retrospectively while FCM-treated patients were recorded prospectively.

**Results:**

A total of 266 patients were included: 111 received FCM (median dose 1000 mg) and 155 were no-IV iron subjects. Both groups were similar in terms of demographic characteristics, tumor location, surgical approach, and intra-operative bleeding severity. The FCM group showed a significant lower need for red blood cell (RBC) transfusion during the study (9.9 vs. 38.7 %; OR: 5.9, *p* < 0.001). In spite of lower hemoglobin levels at baseline diagnosis and lower transfusion rates in the FCM group, the proportion of responders was significantly higher with respect to the no-IV group both at hospital admission (48.1 vs. 20.0 %, *p* < 0.0001) and at 30 days post-surgery (80.0 vs. 48.9 %, *p* < 0.0001). The percentage of patients with normalized hemoglobin levels was also higher in the FCM group (40.0 vs. 26.7 % at 30 days, *p* < 0.05). A lower number of reinterventions and post-surgery complications were seen in the FCM group (20.7 vs. 26.5 %; *p* = 0.311). The FCM group presented a significant shorter hospital stay (8.4 ± 6.8 vs. 10.9 ± 12.4 days to discharge; *p* < 0.001).

**Conclusions:**

Preoperative ferric carboxymaltose treatment in patients with CC and iron deficiency anemia significantly reduced RBC transfusion requirements and hospital length of stay, reaching higher response rates and percentages of normalized hemoglobin levels both at hospital admission and 30 days post-surgery.

## Introduction

A large number of different tumors occur associated with anemia, which ranges from 25–75 % in cancer patients who undergo surgery [1]. Although differing by tumor location, the overall prevalence of anemia in colon cancer was around 48 % with moderate to severe anemia found in more than 20 % of cases [2]. The etiology of anemia in cancer patients is typically multifactorial, mainly caused by systemic inflammation with increased hepcidin levels promoted by the tumor itself as well as by iron deficiency due to gastrointestinal blood loss that occurs in association with tumor ulceration and with malnutrition derived from the disease itself [3, 4]. Besides, blood losses can be stressed during the surgery.

Preoperative anemia is emerging as a common and important health problem [5]. This condition is extremely common before surgery associated with colorectal cancer (up to 70 % of patients) [6, 7] and has also been demonstrated in association with increased postoperative morbi-mortality and duration of hospitalization as well as with reduced quality of life [5, 7–10]. Anemia might contribute to complications during and post-surgery [6, 7, 9]. In addition, a low preoperative hemoglobin concentration is one of the major risk factors for transfusion in surgery with moderate to high blood losses [5, 6, 10]. Jointly, the perioperative transfusions unfavorably affect patient outcomes and highlight the risk of postoperative infections, surgical reintervention, recurrence-metastasis, and subsequent cancer-related mortality in colorectal cancer surgery [6, 11, 12]. Therefore, in the context of elective surgery, it is advisable as far as possible to detect and evaluate preoperative anemia early enough to start a suitable treatment.

Anemia in this patient population is largely due to absolute or functional iron deficiency. As such, administering iron prior to surgery is considered an appropriate treatment [13, 14]. Most anemic patients are treated with oral iron, although this therapeutic procedure is slow in terms of iron absorption rate and, in the context of a surgical cancer patient, clearly insufficient to restore early enough the hemoglobin levels and for iron deposit repositioning [14, 15]. However, parenteral administration of iron could increase up to five times the erythropoietic response, which is also associated with a lower frequency of adverse effects in comparison with blood transfusions [13, 16, 17].

Previous studies have shown that treatment with intravenous iron administered at least 1 week before surgery increases hemoglobin levels and, consequently, should reduce the need for transfusion of red blood cell (RBC) units during the perioperative period [18–20]. Therefore, the implementation of intravenous iron administration protocols appears to be an effective and safe strategy for the treatment of preoperative anemia and possibly to reduce transfusion requirements and hospitalization in patients scheduled for elective surgery while meeting cost-effectiveness criteria, whenever an early detection and diagnosis of preoperative anemia is achieved with the objective to implement this therapeutic choice [13, 14, 21]. Many unsustained misconceptions constitute a barrier to this approach [15].

With this background in mind and considering the lack of studies with a significant number of patients with colon cancer (CC), this study was designed to evaluate the efficacy of a preoperative administration protocol of intravenous (IV) ferric carboxymaltose (FCM) in colon cancer patients with iron deficiency anemia. Evaluation was completed by assessing the relative reduction in RBC transfusion requirements, post-surgery complications (1 month after surgery), and the total length of hospital stay compared with a retrospective cohort of patients that had not received IV iron.

## Material and methods

This is a non-interventional study conducted in Spain as a multicenter survey involving two cohorts of consecutive patients with colon cancer and iron deficiency anemia. At diagnosis, the comparator group with no-IV iron treatment (no-IV iron group) was obtained retrospectively while patients treated with ferric carboxymaltose (FCM group) (Ferinject®; Vifor Pharma España S.L.) were recorded prospectively.

Inclusion of prospective patients was completed between February 2012 and September 2012 in a specialized hospital setting at 11 Spanish hospitals. In the same participant centers, the retrospective cohort was obtained in a sequential manner from surgical intervention 2011 registries and independently of outcomes. All subjects gave their informed consent prior to their inclusion in the study. Approval by the appropriate Institutional Review Boards was obtained.

The study population in both groups included patients aged 18 years or over, diagnosed with colon adenocarcinoma located at least 15 cm above the anal margin, with elective surgery programmed under curative purposes. Iron deficiency anemia was defined according to WHO criteria (hemoglobin (Hb) <13 g/dL in men and <12 g/dL in women) [22], serum ferritin <30 ng/mL, and/or transferrin saturation index <20 %. There were no restrictions on the surgical approach (laparoscopy, open surgery, single port, etc.). The study excluded patients who had rectal neoplasms, emergency or palliative surgery, other linked illnesses associated with anemia such as renal failure or hematological syndromes, tumor recurrence, or a clinical history of blood transfusions during the past 30 days.

The primary outcome of the study was the relative reduction in perioperative and at 30-day postoperative allogenic RBC transfusion requirements. The secondary end points included the reduction of hospital length of stay (or time to discharge), the incidence of postoperative complications registered during the first month after surgery, the evolution of hemoglobin and iron parameters during the study period, and the proportion of patients with normalized Hb levels and response rate.

The physicians responsible for the patients’ care, surgery, hospital discharge, and follow-up were unaware of study interventions. When the center diagnosed a patient with colon cancer and concurrent iron deficiency anemia, and who met all the selection criteria, the investigator requested the inclusion of the patient in the program of IV iron with ferric carboxymaltose administration. The total dose was obtained using the ferric carboxymaltose product information dosing scheme [16, 17]. Whenever possible, the FCM administration was between 2 and 4 weeks before the scheduled surgery.

As standardized hospital clinical practice, a complete blood test was also performed at diagnosis, the day of admission for surgery, at discharge, and at 30 days post-surgery. Peri- and postoperative RBC transfusions were based on the pertinent blood tests and as in hospital clinical practice: being always performed in patients with hemoglobin levels under 7 g/dL, under physician criteria between 7 and 9 mg/dL, and not recommended over 9 g/dL. Normalized Hb levels were established as ≥12 g/dL in women and ≥13 g/dL in men [22]. Patient response was considered when Hb increases ≥1.5 g/dL.

Based on the primary end point, the calculated sample size of the study was approximately 111 patients per group. This estimated size allows the detection of at least a 20 % reduction in RBC transfusion requirements with a statistical power of 90 % and using a 95 % confidence interval (CI). Continuous end points were summarized using descriptive statistics: *n*, mean, standard deviation, minimum, median and maximum, 95 % CI, and number of missing observations. For discrete end points, the frequency and percentage for each response category were calculated as well as 95 % CI and number of missing data. Missing data was excluded when calculating percentages relative to the total sample. Student *t* test or Mann-Whitney U test were used depending on distribution of variables.

## Results

### Baseline and clinical characteristics

Baseline characteristics were similar for both cohorts studied (Table 1). In total, 266 patients were included: 111 received FCM (57.3 % males, mean age 72.9 ± 11.1) and 155 were in the no-IV iron group (55.8 % males, mean age 70.8 ± 10.3). Both groups were similar in terms of tumor location, medical history, and surgical approach and procedures, as well as the proportion of patients with moderate or heavy blood loss (≥50 mL) during surgery (Table 1). Anemia at diagnosis was more pronounced in the FCM group.

**Table 1:** Baseline, clinical, and surgical characteristics of the patients

	No-IV iron	FCM	*p*
Number, *N*	155	111	
Sex, male (%)	55.8	57.3	0.817
Age, years (mean ± SD)	70.8 ± 10.3	72.9 ± 11.1	0.121
BMI, kg/m^2^ (mean ± SD)	28.2 ± 4.9	27.7 ± 5.7	0.429
ASA (%)			0.088
I	11.6	5.6	
II	42.2	56.5	
III	44.2	35.2	
IV	2.0	2.8	
Past medical history (%)			
Diabetes	35.7	29.2	0.276
Hypertension	56.2	56.0	0.968
EPOC	12.3	13.9	0.713
Heart disease	19.1	22.6	0.486
Anticoagulant therapy	7.9	15.7	0.050
Tumor location (%)			0.846
Ascending colon	55.6	59.2	
Transverse colon	9.2	6.8	
Descending colon	8.5	9.7	
Sigmoid colon	26.8	24.3	
Symptomatology (%)			0.181
Rectorrhagia	23.2	27.0	
Obstruction	2.6	2.7	
Pain	16.8	9.9	
Anemic symptoms	43.2	36.9	
None	14.2	23.4	
Iron treatment at diagnosis (%)			
Oral	100.0	15.3	
Ferric carboxymaltose	0.0	100.0	
Surgical approach (%)			0.105
Open surgery	52.6	42.7	
Laparoscopy	47.4	54.5	
Single-port surgery	0.0	0.9	
NOTES	0.0	1.8	
Surgical intervention (%)			0.838
Right hemicolectomy	61.4	64.0	
Sigmoidectomy	24.1	21.0	
Left hemicolectomy	9.0	11.0	
Segmental resection	5.5	4.0	
Intraoperative blood losses (%)			0.857
Low	47.1	43.3	
Moderate (≥50 ml)	44.2	46.3	
High (>250 ml)	8.7	10.4	
Intraoperative moderate/severe blood losses (%)			
Open surgery	62	73	0.356
Laparoscopic	43	39	0.817

*ASA* American Society of Anesthesiologists, *BMI* body mass index, *EPOC* excess post-exercise oxygen consumption, *FCM* ferric carboxymaltose, NOTES

All patients in the retrospective cohort (no-IV iron group) were receiving different doses and formulations of oral iron supplementation at the time of diagnosis. Within the prospective cohort, the median total FCM dose was 1000 mg iron (mean 1275 ± 430.1 mg) and the administration was 28.5 ± 16.7 days before surgery (mean ± SD).

### Primary outcome: transfusion requirements

A significantly lower percentage of patients in the FCM group required allogenic RBC transfusion during the study: 9.9 vs. 38.7 % (OR: 5.9, 95 % CI: 2.9–11.1, *p* < 0.001). This statistically and clinically significant difference was also observed in the individual peri- and post-surgery periods until day 30 and independently of the type of surgical approach (laparoscopic or open surgery) (Table 2). Overall, the mean number of RBC units transfused during the study period was statistically lower in patients treated with FCM (0.2 ± 0.5 vs. 0.8 ± 0.4, *p* < 0.0001) (see Table 2 for peri- and post-surgery periods). When analyzed by surgery, FCM-treated patients received statistically significant lower mean units of RBC as well (Table 2). Throughout the entire study period, 9 % of patients in the no-IV iron group received 4 or more RBC units vs. 0 % in the FCM group (*p* = 0.5817).

**Table 2:** Need for allogenic RBC transfusion (percentage of patients) and mean RBC units transfused

	No-IV iron	FCM	*p*
Need for RBC transfusion (overall), %	38.7	9.9	<0.001
Need for RBC transfusions (peri-surgery period), %	31.8	8.3	<0.001
Need for RBC transfusion (post-surgery until day 30), %	16.7	5.0	0.005
Need for RBC transfusion (by surgery), %			
Open surgery, %	37.7	17.1	<0.05
Laparoscopic, %	25.4	0.0	<0.0001
Mean units of RBC transfused (overall)	0.8	0.2	<0.0001
Mean units of RBC transfused (peri-surgery)	0.5	0.1	<0.0001
Mean units of RBC transfused (post-surgery until day 30)	0.3	0.1	<0.05
Mean units of RBC transfused (by surgery)			
Open surgery	1.0	0.4	<0.01
Laparoscopic	0.6	0.0	<0.0001

*FCM* ferric carboxymaltose, *RBC* red blood cell

### Evolution of hemoglobin and iron metabolism parameters

**Fig. 1:**
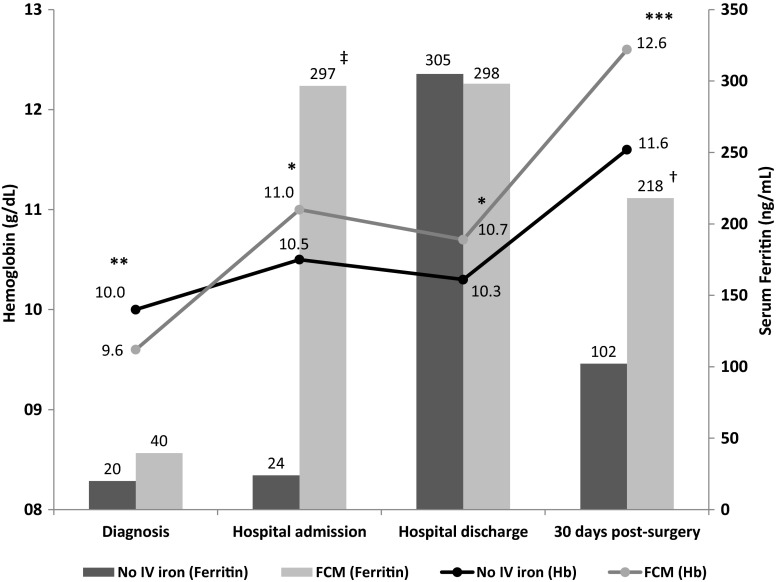
Evolution of hemoglobin levels (g/dL) at four time points: diagnosis, hospital admission, discharge, and 30 days post-surgery. Groups were not censored for transfusions. Significant differences between groups are marked with an *asterisk* for Hb and *dagger* for serum ferritin (*p < 0.05; **^/†^ *p* < 0.005; ***^/‡^ *p* < 0.001). Ferric carboxymaltose (*FCM*) was administered during diagnosis period

All the hematological parameters, except serum ferritin and transferrin saturation index at hospital discharge, presented significant differences between the FCM and no-IV iron groups at admission time point and 1 month after surgery (Table 3, Fig. 1). Importantly, the FCM group received overall less RBC transfusions, and data has not been censored for transfusions.

**Table 3:** Mean (±SD) hemoglobin, hematocrit, MCV, and iron parameters at different time points before and after surgery. Groups were not censored for transfusions

	Hb (g/dL)	Hematocrit (%)	s-Ferritin (ng/mL)	T-SAT index (%)	MCV (10^−15^ L)
Diagnosis					
No-IV iron	10.0 ± 1.2	31.8 ± 3.4	20.0 ± 20.8	7.6 ± 4.9	78.4 ± 8.6
FCM	9.6 ± 1.4**	31.1 ± 3.9	39.6 ± 62.9	8.0 ± 5.9	79.3 ± 8.2**
Hospital admission					
No-IV iron	10.5 ± 1.3	33.1 ± 3.9	24.0 ± 21.6	7.9 ± 3.1	80.6 ± 7.9
FCM	11.0 ± 1.7*	34.9 ± 5.0*	296.6 ± 292.1***	19.1 ± 11.6***	84.5 ± 7.3***
Hospital discharge					
No-IV iron	10.3 ± 1.2	32.1 ± 3.5	305.0 ± 323.6	16.9 ± 15.4	82.6 ± 6.9
FCM	10.7 ± 1.4*	33.4 ± 4.4*	298.1 ± 294.5	18.2 ± 10.5	86.4 ± 6.6***
30 days post-surgery					
No-IV iron	11.6 ± 1.3	36.4 ± 5.9	102.1 ± 210.1	15.9 ± 13.3	83.0 ± 7.4
FCM	12.6 ± 1.3***	38.8 ± 3.7***	218.1 ± 218.3*	25.1 ± 18.2*	88.6 ± 5.3***

*FCM* ferric carboxymaltose, *s-ferritin* serum ferritin, *T-SAT* transferrin saturation index (%), *MCV* mean corpuscular volume (10^−15^ L)

*no-IV iron vs. FCM, *p* < 0.05; **no-IV iron vs. FCM, *p* < 0.005; ***no-IV iron vs. FCM, *p* < 0.001

Figure 1 reflects the evolution of hemoglobin levels in both groups at the four study-defined time points. Despite the lower levels of hemoglobin at diagnosis for the FCM group (9.6 ± 1.4 vs. 10.0 ± 1.2 g/dL, *p* < 0.005), and much lower RBC transfusion rate, significantly higher hemoglobin concentrations were achieved by the FCM group at hospital admission, discharge, and 30 days post-surgery (Table 3; Fig. 1). Mean total hemoglobin increases significantly favor the FCM group between diagnosis and hospital admission (1.5 vs. 0.5 g/dL; *p* < 0.0001) and between diagnosis and 30 days post-surgery (3.1 vs. 1.5 g/dL; *p* < 0.0001). This was also the case when doing the analysis for transfused and non-transfused patients: mean total hemoglobin increase between diagnosis and 30 days post-surgery in transfused patients was 3.5 ± 1.9 FCM vs. 1.4 ± 1.6 no-IV (*p* < 0.05) and in non-transfused patients was 3.1 ± 1.9 FCM vs. 1. 6 ± 1.8 no-IV (*p* < 0.001).

A similar percentage of patients with Hb ≤ 10 g/dL was observed at diagnosis (79 %) in both groups. This rate descends by nearly 20 % for the no-IV group and 30 % in the FCM group at time of hospital admission. The percentage of patients with Hb ≤ 10 g/dL was significantly lower in the FCM group at hospital discharge (61.6 % FCM vs. 75.7 % no-IV iron, *p* < 0.05) and at 30 days post-surgery (12.0 % FCM group vs. 28.9 % no-IV iron, *p* < 0.05). Furthermore, the percentage of patients with normalized hemoglobin at 30 days post-surgery was significantly higher in the FCM group vs. no-IV iron (40.0 vs. 26.7 %, *p* < 0.05).

Figure 1 also shows the evolution of serum ferritin levels. At 30 days after-surgery, the average FCM-treated patient presented no recognizable signs of iron deficiency anemia (being the mean of Hb: 12.6 g/dL [≥12 g/dL]; serum ferritin: 218 ng/mL [≥30 ng/mL]; and saturation transferrin index: 25.1 % [≥20 %]) compared with the no-IV group that did not reach normalized mean values (Table 3).

**Fig. 2:**
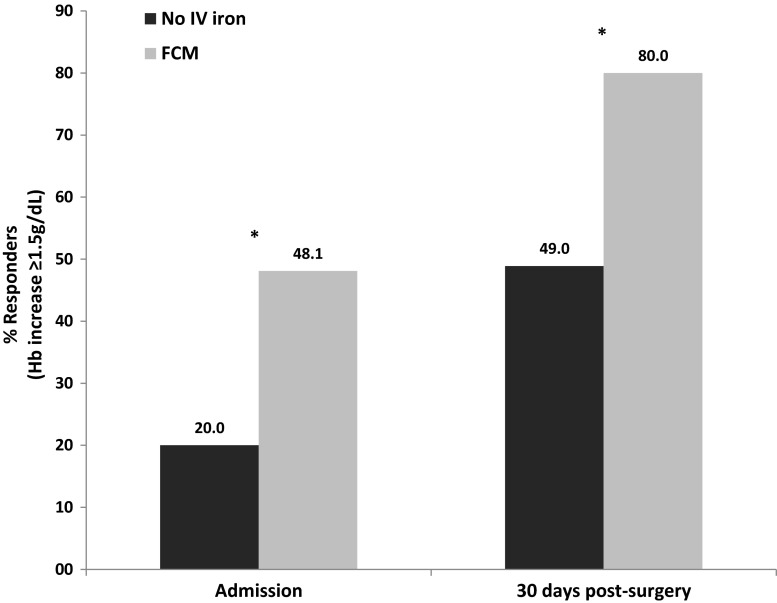
Percentage of hemoglobin responders—defined as those with an Hb increase of ≥1.5 g/dL—at hospital admission and 30 days post-surgery with respect to Hb diagnosis levels. Data was not censored for transfusions

Figure 2 illustrates how the percentage of hemoglobin responders (Hb increase of ≥1.5 g/dL) significantly increased in the group treated with FCM compared to the no-IV group: 48.1 vs. 20.0 % between diagnosis and hospital admission (*p* < 0.0001) and 80.0 vs. 48.9 % between diagnosis and 30 days after surgery (*p* < 0.0001).

### Post-surgery complications

A numerically lower total number of reinterventions and complications related to surgery (including suture dehiscence, paralytic ileus, hemoperitoneum, rectal bleeding, thromboembolism, etc.) at 30 days after surgery were seen in the FCM group in comparison with no-IV patients: 20.7 vs. 26.5 % (OR = 1.4; 95 % CI: 0.8–2.4; *p* = 0.311) (Table 4).

**Table 4:** Incidence of post-surgery complications

	No-IV iron (*n* = 155)	FCM (*n* = 111)	*p*
Total number of complications (from surgery until 30 days post-surgery) (% patients)	25.5	22.5	NS
Infection	48.6	56.5	
Suture failure	32.4	21.7	
Paralytic ileus	24.3	17.4	
Rectorrhagia	16.2	8.7	
Hemoperitoneum	10.8	4.3	
Thromboembolic complication	10.8	0.0	
Surgical reintervention (% patients)	13.4	6.7	NS
Hospital readmission (surgical-related cause) (% patients)	3.9	4.0	NS

*FCM* ferric carboxymaltose, *NS* non-significant

### Impact on hospital stay

**Fig. 3:**
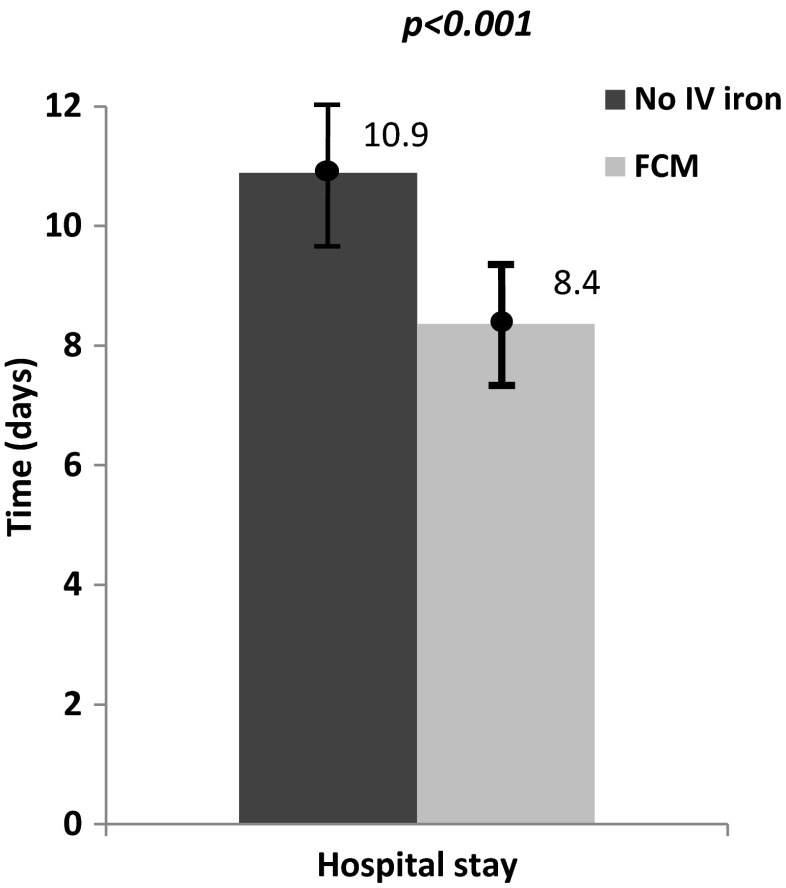
Mean length of hospital stay measured from the day of surgery until the hospital discharge

The length of hospital stay, measured from the day of surgery until the day of discharge, is shown in Fig. 3. The FCM group had a significantly shorter mean length of hospital stay: 8.4 ± 6.8 days compared to the no-IV iron group (10.9 ± 12.4 days) (*p* < 0.001). Overall, patients that required RBC transfusion had a longer hospital stay than patients without transfusion (FCM 9.2 ± 9.4 days vs. no-IV iron 12.0 ± 13.0 days; *p* < 0.005).

### Safety of ferric carboxymaltose

From the FCM-treated patients, no deaths, hypersensitivity, or other serious adverse drug reactions were observed.

## Discussion

Preoperative anemia in patients with surgical bleeding risk associated with colorectal cancer has proved very prevalent and is an independent risk factor for the requirements of allogeneic RBC transfusion [12]. Under this global perspective, and considering the lack of controlled studies with a significant number of patients, our prospectively defined and treated population demonstrated a significant clinical benefit for the preoperative administration of an IV iron supplementation using ferric carboxymaltose (FCM) when compared to a retrospective cohort (with similar baseline characteristics). This overall fourfold reduction of transfusions (and hence associated risk factors) was achieved using FCM which was well tolerated in this group of patients and demonstrated a favorable benefit-risk profile. Furthermore, the observed needs for transfusion were five times (pre-and intraoperative) and almost three times higher (postoperative) for the group that had not received IV iron. Interestingly, the patients in this cohort were all receiving oral iron supplementation at time of diagnosis.

Similar decreases in transfusion requirements for FCM-treated patients have been reported in a study that compares the pre-operative administration of FCM with the IV iron sucrose administration in a small cohort of 45 patients treated for anemia in colon cancer resection [18]. In this study, Bisbe and collaborators observed a noticeable reduction in the percentage of patients that required RBC transfusion in the FCM group (from 40 % with iron sucrose to 7 % with FCM), as well as fewer iron administration sessions (as FCM may be administered at a single dose of 1000 mg iron per session vs. only 200 mg for iron sucrose). Analogously, similar trends in reducing allogenic RBC transfusion requirements were observed with FCM in a three-cohort retrospective study that included a total number of 154 patients with GI cancer submitted to laparoscopic resection (gastrectomy, right or left colectomy, or rectum resection) [23]. Even though initial levels of Hb were higher in the non-anemic group of patients, the anemic FCM group required similar mean RBC units transfused, and both were significantly lower (*p* < 0.001) than in the anemic no-IV-treated group (0.5, 0.4, and 2.4, respectively).

In contrast, a previous randomized placebo-control clinical trial (*n* = 60) did not find support for the use of intravenous iron sucrose (600 mg iron in two divided doses, 14 days before surgery) as a preoperative supplementation to reduce the likelihood of allogenic RBC transfusion for patients undergoing resectional surgery for colorectal cancer (19.2 % placebo vs. 5.9 % in iron sucrose; *p* = 0.335) [24]. However, although not reaching statistical significance, the number of transfusions was still numerically higher in the placebo group, and it might be hypothesized that the administered iron doses were not sufficient to meet the iron deficit in these patients.

Within our study, and even despite significantly fewer patients transfused, at 1 month post-surgery, as overall, the FCM-treated patients did not reflect signs of anemia, as well as no signs of iron deficiency (mean ferritin and transferrin saturation index were at normal values). Our data in a large patient group extends to the 30-day post-surgery period the previously found evidence for improved hemoglobin concentrations and reduced transfusions in gastrointestinal cancer patients with anemia treated with FCM [23]. Moreover, in those anemic patients included in our study who have received FCM, almost one out of two reached normalized hemoglobin levels after 1 month from the colon cancer resection and despite their lower mean hemoglobin levels at diagnosis and their lower rate of transfusion requirement, while in patients with no-IV iron administration, this percentage was only around 26 %. Our data further supports the preoperative treatment with ferric carboxymaltose in anemic colon cancer patients who are planned for surgery, with benefits extending until 30 days post-surgery.

In addition to the reduction in transfusion requirements and the improved iron parameters, our study demonstrates for the first time the benefit of a preoperative FCM administration strategy in the peri- and post-surgery periods when treating this kind of patients. Specifically, importance should be given to the observed relation to the mean hospital length of stay which was significantly reduced with the FCM administration by a mean total of 2.5 days. Likewise, the postoperative benefits of intravenous iron administered previously to the surgery have been reported recently in other patient profiles, i.e., those submitted to non-cardiac surgery, gynecological tumor resection, cardiac valve replacement, and orthopedic procedures in terms of reduction of post-surgery complications and reduced hospital length of stay [10, 16, 19, 20, 25]. Finally, it has ruled out any link between the surgical approach and possible differences between groups with regard to complications in the 30-day post-surgery period.

As a result of these improvements, pretreatment with FCM in anemic colon cancer patients seems to be a cost-effective intervention. The incremental costs of IV iron treatment are very likely to be offset by the cost savings due to reductions in length of stay and transfusion rates. The extent of this economic advantage will be assessed in a further within-trial analysis. In a previous Spanish cost analysis taking into account both drug acquisition costs of FCM and iron sucrose as well as administration costs in anemic patients undergoing major elective surgery, the final cost benefit of FCM administration per treatment compared with the common IV iron sucrose therapy was demonstrated by providing a mean of €63 savings per patient FCM treatment [18].

As strengths of our study, it should be mentioned the number of patients considered for the analysis in the predefined groups as well as their homogenous baseline and clinical characteristics in both groups. It is also important to highlight that the significant trends observed in the measured outcomes were achieved after similar laboratory values at diagnosis and surgical characteristics in both groups, although the FCM group showed a slightly reduced hemoglobin at baseline. The present study has several limitations that warrant acknowledgment. Specifically, the non-randomized design limits the interpretation of the results. However, these patients represent “real-life” clinical practice, and hence, this is also a strength of this research. Future randomized controlled trial(s) focused on the use of FCM treatment in preoperative anemic colorectal cancer patients vs. a similar active supplement may aid to confirm our findings.

In conclusion, our data demonstrate that preoperative ferric carboxymaltose treatment in iron-deficient colon cancer patients with anemia significantly reduced the length of hospitalization, decreased both the perioperative and postoperative allogenic RBC transfusion requirements, showed no signs of iron deficiency anemia at 30 days post-surgery, and was safe and well tolerated. Based on reduction of RBC transfusions and length of hospital stay, it can be assumed that the preoperative administration of ferric carboxymaltose in iron-deficient colon cancer patients with anemia undergoing surgery could result in significant cost savings.
